# Cutaneous Vesicular of COVID-19 in Two Burn Patients

**DOI:** 10.29252/wjps.9.3.331

**Published:** 2020-09

**Authors:** Abdolkhalegh Keshavarzi, Ali Akbar Mohammadi, Mehdi Ayaz, Fatemeh Javanmardi, Mohammad Ali Hoghoughi, Babak Shirazi Yeganeh, Amir Emami, Mandana Mackie, Rahimeh Akrami, Sorayya Iranpak

**Affiliations:** 1 Department of Surgery, Shiraz University of Medical Sciences, Shiraz, Iran;; 2 Burn and Wound Healing Research Center, Shiraz University of Medical Sciences, Shiraz, Iran;; 3 Department of Plastic and Reconstructive Surgery, Shiraz University of Medical Sciences, Shiraz, Iran;; 4 Division of Burn and Reconstructive Surgery, Department of Surgery, Shiraz University of Medical Sciences, Shiraz, Iran;; 5 Pathology Department, Shiraz University of Medical Sciences, Shiraz, Iran;; 6 Department of Microbiology, Shiraz University of Medical Sciences, Shiraz, Iran

**Keywords:** Burn, Vesicle, Blister, COVID-19, SARS-CoV-2

## Abstract

Despite the whole world’s effort for controlling an ongoing global outbreak caused by new corona virus; it is still a major public health issue. Any hospitalized patient or outpatient in burn departments should be considered as a potential infectious source of COVID-19, which may cause an overwhelming of disease. However, there are no previous experiences about COVID-19 in burn patients all over the world, and here we reported two burn cases at Amir-al-Momenin Burn Hospital Affiliated to Shiraz University of Medical Sciences, Shiraz, Iran with skin manifestations, which were detected as a rarely COVID-19 symptom. A 13-year-old girl [total body surface area (TBSA): 18%] and a 37-year-old woman (TBSA: 30%) who had burn injuries by gas explosion and car accident, respectively were enrolled. After admission, some vesicular injuries were visible in burn area. To confirm, skin biopsy specimens were either sent for histopathology examination or for real time polymerase chain reaction (PCR) as follow: Herpes Simplex Virus (HSV), chicken pox, and potassium hydroxide (KOH) for fungal infections. All test results were negative. Although they had no symptoms of COVID-19, two swabs from nasopharyngeal and oropharyngeal samplings were taken, the result was negative either. Specimens were obtained from vesicular lesions for qRT-PCR assay of COVID-19. According to the molecular results for vesicular samples, all the results were positive for COVID-19. Unlike all other COVID-19 patients who have respiratory symptoms, SARS-COV-2 appeared by cutaneous vesicular and blisters in two burn cases.

## INTRODUCTION

An outbreak of pneumonia with a unknown cause began in the Wuhan, China at the end of 2019, and the disease inexorably spread and created a global concern.^1^ According to the initial investigations, it was shown that the disease retrieved from a novel Corona virus and was named severe acute respiratory syndrome Corona virus 2 (SARS-CoV-2).^2^ This contagious viral disease (Corona virus disease; COVID-19) caused a respiratory infection that has a protracted clinical course and may result in an increased inflammatory acute respiratory distress in patients; usually after two weeks.^3^ Although there are a lot of ambiguities and uncertainties about COVID-19; but immunosuppressed people were found to be more in danger of infection complications.^4^

One of the high risk immunosuppressed hospitalized patient groups in the world are burn victims whom in general, are susceptible to every kind of infection, while their susceptibility to COVID-19 is not clear, but they are more likely at risk for severe illnesses, such as respiratory viral infections.^5^ Burn patients according to their conditions such as loosing skin as the first line of defense, specific nutritional regimen, use of different medications, and somehow related complications such as chronic liver and renal failures, make this group deliberately weaker. These conditions make burn victims be more susceptible and expose to serious infections, which may threaten them by different patterns of infections and different kinds of symptoms may appear.^6^

Sensitivities in burn patients’ care, and the outbreak of recent pandemic (COVID-19) had a large impact on our healthcare system and infection control strategies in this group of victims in Iran. Despite all considerations and emphasis on infection control strategies in our burn center, we have two burn cases with skin blisters at Amir-al-Momenin Burn Hospital affiliated to Shiraz University of Medical Sciences, Shiraz, Iran. However, there are no previous experiences about COVID-19 in burn patients all over the world, and here we reported two burn cases with skin manifestations, blisters, and vesicular injuries, which were detected as rare COVID-19 symptoms. This study was approved by Shiraz University of Medical sciences by ethical code of IR.SUMS.REC. 1399.022.

## CASE REPORT

CASE 1

A 13-year-old girl was admitted in intensive care unit (ICU) in Amir-al-Momenin burn Hospital. She was burned due to gas leak and explosion at home in March 17, 2020. She suffered from deep 2^nd^ degree burns on face and neck, and full thickness burn on both forearms, feet and thighs. Total burn surface area (TBSA) was estimated 18 percent. In order to release the pressure over the involved deeper tissues and to restore their circulation, escharotomy was done on back of right hand, a day after admission. 

The patient had undergone a treatment, with debridement of the burned tissue on both hands, 8 days after burn injury (March 24, 2020). Moreover, skin graft with spilt thickness graft (STSG) was implemented 12 days after admission (March 28, 2020). The fluid resuscitation was administered based on the Parkland formula and it was monitored in relation to urine output. Routinely, exfoliative and quantitative samplings were performed for wounds, urine and hemo-cultures. 

**Fig. 1:**
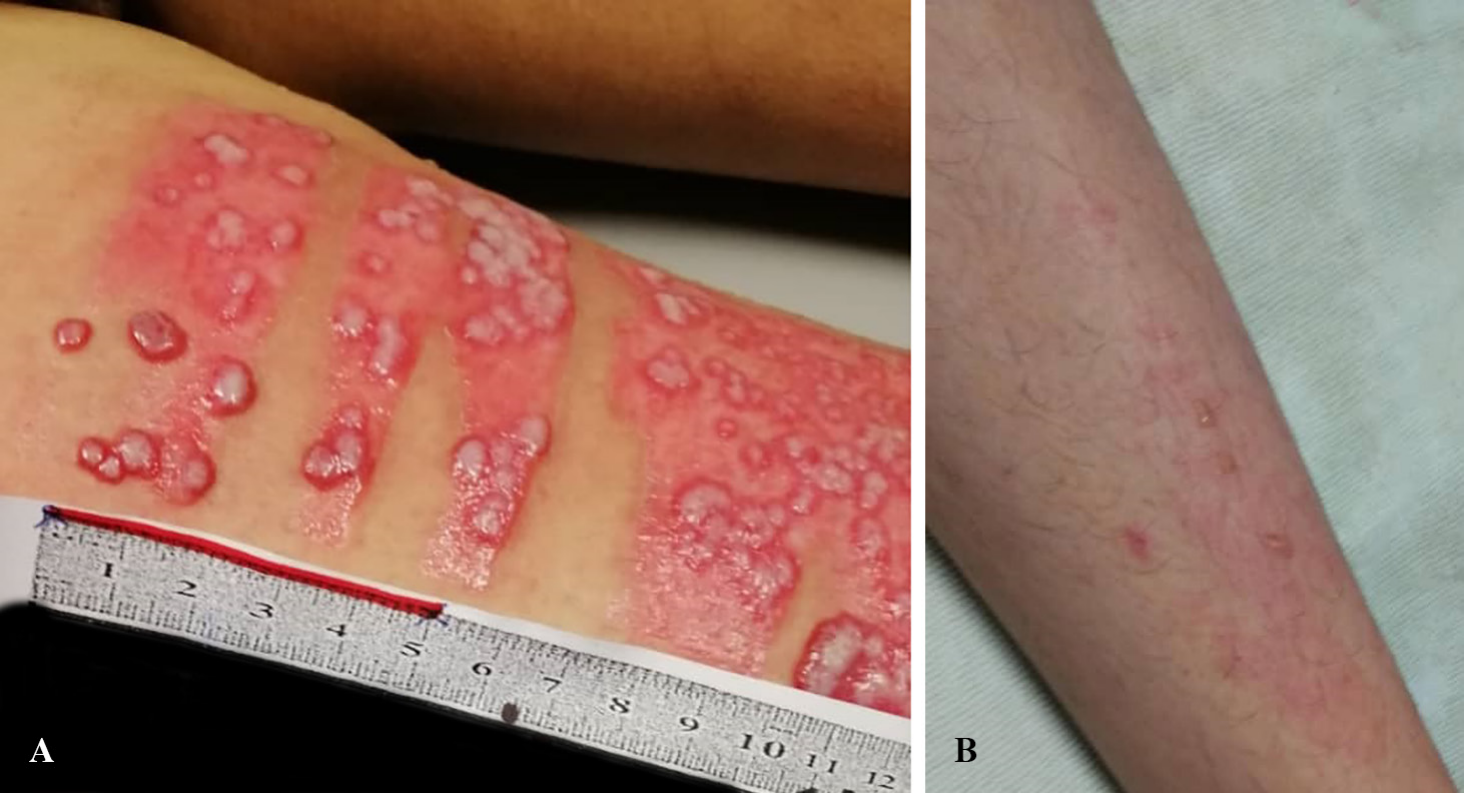
**A: **Case 1: Vesicular manifestation on burn area/5^th^ April 2020. **B: **Case 1: Vesicular manifestation areas other than burns/27^th^ April 2020

Coagulase-negative staphylococci (CoNS) was isolated in urine culture in the first day of admission, so antibiotic therapy was undertaken by cefazolin (500, QID) based on sensitive antibiogram test. No microorganism growth was seen in hemo-culture, urine and wound cultures too. Beside the standard treatment, the potassium permanganate was applied once daily to the entire surface area of the ulcer. In the mentioned center, regularly changing dress was performed daily for all patients. For the mentioned case, some vesicular injuries were noted in the thigh of injured zone 20 days post-burn (April 5, 2020) and in other injured areas too (Figure 1).

The patient was immediately isolated and quarantined in a room with related guidelines and sterilization processes based on infection control strategies. Two days after onset of vesicular injuries, *Pseudomonas aeruginosa* was isolated from wounds. Although some antibiotics were administered for the patient based on her condition (Table 1); other topical antibiotics, such as tetracycline and fucidin were commenced for covering the wounds during dressing. Following blisters manifestations and receipt of antibiogram results for wound infection, previous antibiotics were stopped, and vancomycin (QID), colistin (TID), acyclovir (QID), and caspofungin were administered. 

**Table 1:** Timeline of disease course according to days from hospitalization till 26^th^ March. Abbreviation: U/C: Urine culture, W/C: Wound culture, STSG: Split thickness skin graft

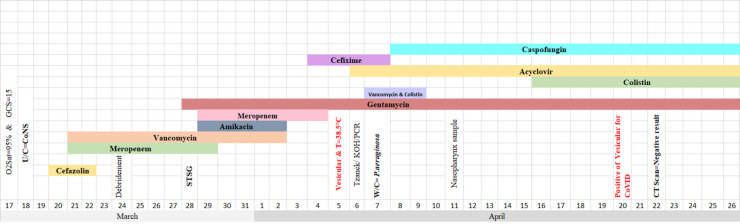

**Fig. 2:**
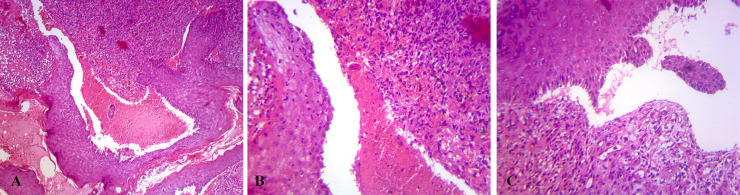
**A:** Subepidermal bullae containing RBCs and a few WBCs (H&E, 40x). **B:** Subepidermal bullae containing RBCs and a few WBCs. Dermis demonstrate granulation tissue with prominent lymphocyte infiltration (H&E, 100×). **C:** Subepidermal bulla with underlying lymphocyte rich granulation tissue (H&E, 100×).

Based on the initial hypothesis and to resolve the misidentification of bacteria or virus, several specimens were obtained from vesicular lesions with sterile needle or scalp within the first days after appearance. For primary evaluation, Tzanck test was implemented assuming a Herpes infection. For further confidence and dispel doubts; skin biopsy specimens were either sent for histopathology examination (Figure 2) or real time polymerase chain reaction (PCR) as follows: Herpes Simplex Virus (HSV), chicken pox, and KOH for fungal infections. The results were negative for all tests. It is worth to mention that IgM and IgG values for HSV were 0.6 and 6.1, respectively. These results showed that the patient was immune against HSV.

Since the patient was hospitalized in pandemic time of COVID-19, she was asked for history of contact with positive infected cases, history of travel within last two weeks and any underlying diseases. None of them were true based on her and her parent’s declaration. Although no abnormality was observed in the vital signs and no symptoms of cough, fatigue or shortness of breath was seen, but the upper respiratory (two swabs from nasopharyngeal and oropharyngeal; NP/OP, taken in one tube) swab sampling was taken for assurance on day 26^th^ of admission (April 11, 2020) to test for COVID-19. 

According to the qRT-PCR reports for NP/OP samples, the result was negative for COVID-19. According to the sensitivity of chest CT imaging (97%) as a reliable, feasible, and rapid method to diagnose and assess COVID-19, this method was implemented (April 22, 2020) based on the radiology results and no evidence of COVID-19 was seen on images. Following treatment of the patient, topical acyclovir was used for 3 days, but in absence of any improvement, it was changed to tetracycline. Laboratory test results before and after skin manifestations were presented in Table 2. 

**Table 2:** Laboratory findings of cases during the hospitalization

**Biomarkers**	**Before manifestation**	**After manifestation**
**Case 1 (13 years old)**		
White blood cells (µL) (Min, Max)	(4600, 14300)	(500, 9500)
Hemoglobin (g/dl) (Min, Max)	( 7.3, 15.2)	(6.9, 11.1)
Platelets (µL) (Min, Max)	(165000, 474000)	(319000, 329000)
Creatinine phosphokinase (U/dl)	(108, 129)	29
ESR (mm/1h) (Min, Max)	(2, 50)	(20, 22)
CRP	Negative	(1+, 3+)
Lactate dehydrogenase (U/L) (Min, Max)	(171, 340)	(301)
PCT (Min, Max)	(<0.05, 0.28)	0.07
Albumin (g/L) (Min, Max)	(2.4, 4.4)	(2, 3.3)
Aspartate aminotransferase (U/L) (Min, Max)	(13, 58)	(14, 16)
Aspartate aminotransferase (U/L) (Min, Max)	(11,51)	(12, 17)
**Case 2 (38 years old)**		
White blood cells (µL) (Min, Max)	(5800, 15800)	(4000, 12600)
Hemoglobin (g/dl) (Min, Max)	(6.7, 10.1)	(8, 10.6)
Platelets (µL) (Min, Max)	(493000, 967000)	(228000, 617000)
Creatinine Phosphokinase (U/dl)		
ESR (mm/1h) (Min, Max)	(89, 130)	(85, 110)
CRP	(Negative, 1+, Trace)	(1+, Trace)
Lactate dehydrogenase (U/L) (Min, Max)	485	364
PCT (Min, Max)	(< 0.01, 0.15)	0.1
Albumin (g/L) (Min, Max)	(2.1, 2.8)	(2.8, 3.4)
Aspartate aminotransferase (U/L) (Min, Max)	(29, 49)	(13, 25)
Aspartate aminotransferase (U/L) (Min, Max)	(27, 41)	(10, 24)

Overall, burn treatment strategies and critical care were continued. So by considering all of the laboratory results with full consultation, a few specimens were obtained from vesicular lesions using a sterile needle (one needle-one sample) for qRT-PCR assay of COVID-19 on day 35^th^ of the admission (April 20, 2020). According to the molecular **results for** vesicular samples**, all the results were positive for COVID-19. **Altogether, the patient was hospitalized for 40 days (April 26, 2020). Although she had no common respiratory symptoms of COVID-19, surgical mask and other respiratory protections were introduced and applied for her. 

Figure 1A shows the vesicles surrounded by erythema in the burned area. The vesicles formed in areas other than burn regions (Figure 1B). To ensure that the viral infection is not transmitted through hospital personnel, and according to the safety protocols of recent pandemic, all of the ward staffs were tested for COVID-19. In this evaluation, two personnel were detected as a carrier for COVID-19 and were immediately banned to continue their activities in the hospital and quarantine. 

CASE 2

A 38-year-old woman was transferred from Shahid Rajaee Trauma Hospital to Amir-al-Momenin Burn Hospital (March 22, 2020). She had bone fractures and burn injuries due to a car accident on March 14, 2020. Because of fractures, she was first treated for 8 days at mentioned trauma center. In the arrival to the burn center, she was conscious, and the vital signs were normal, but she had a cough with sputum, moreover, according to the trauma hospital report, she had right lung contusion too. The injury caused full-thickness burns in both forearms, both thighs, one foot, and the whole face. 

The corporal burn surface was estimated 30 percent for this case. Empirical systemic antibiotic therapy was begun using meropenem in the admission day (Table 3). According to the results of wound culture (March 23, 2020), heavy growth of *P. aeruginosa* was detected. Based on the antibiogram results, the isolates were resistant to amikacin, ceftazidime, ciptoflxocin, gentamycin, piperacillin, and co-trimoxazole and only sensitive to colistin. Urine culture did not show any infection. 

**Table 3:** Timeline of disease course according to days from hospitalization till 26^th^ March. Abbreviation: U/C: Urine culture, W/C: Wound Culture, Sp/C: Sputume culture, STSG: Split thickness skin graft, D &G: Debredement and graft

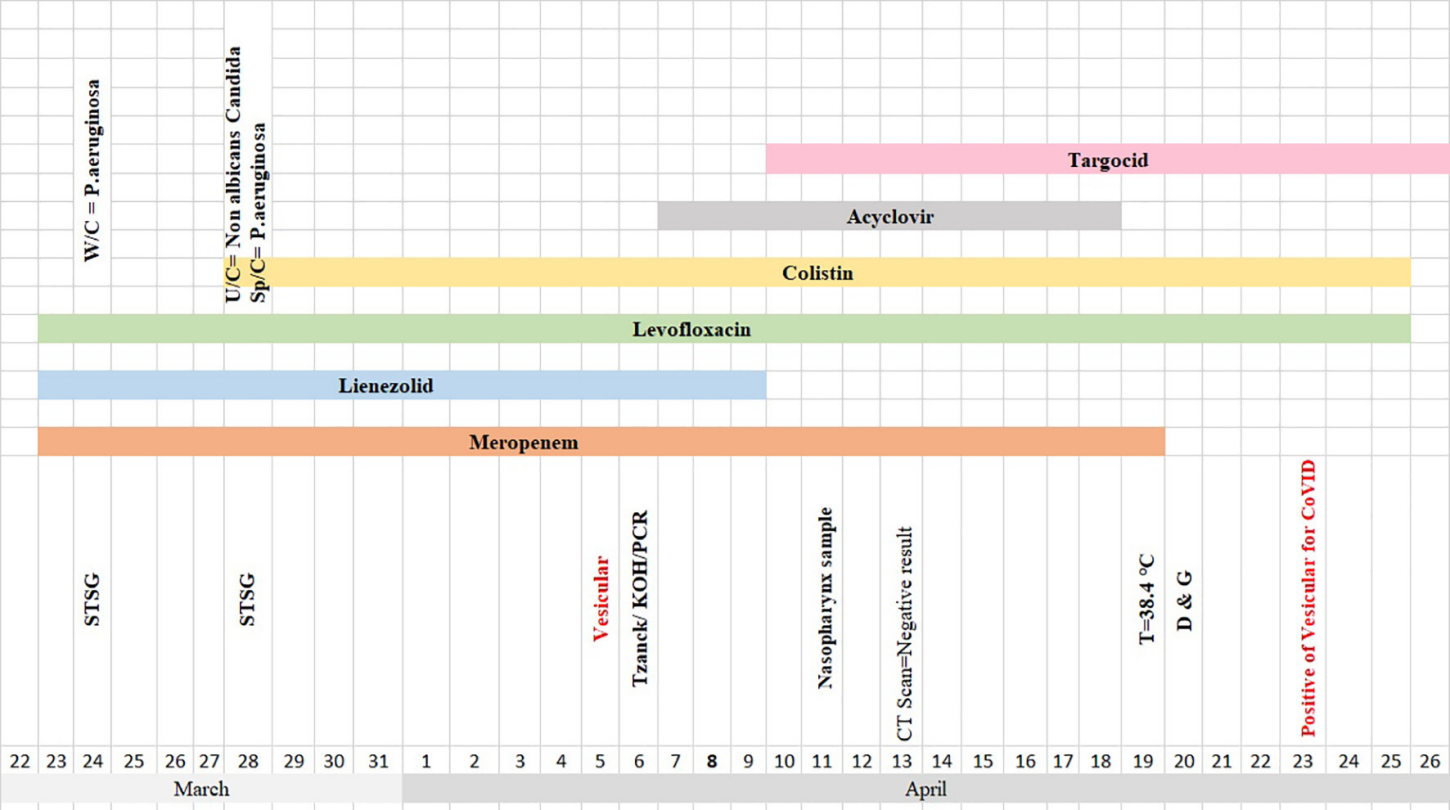

The patient had undergone a treatment of skin graft on the right thigh, leg and also use of amnion dressing on the donor sites (March 24, 2020). Similar to other burn patients; blood, urine and tissue cultures were taken regularly and appropriate antibiotics were prescribed based on the antibiogram results. Six days after admission (March 28, 2020), urine culture was positive for *Candida *non*-albicans* and sputum culture was positive for* P. *a*eruginosa* with the following patterns: Sensitive to colistin, and resistant to amikacin, ceftazidime, ciprofloxacin, co-trimoxazole. Due to the results, the colistin (TID) was prescribed for three days beside the linezolid, and levofloxacin was discontinued. 

**Fig. 3:**
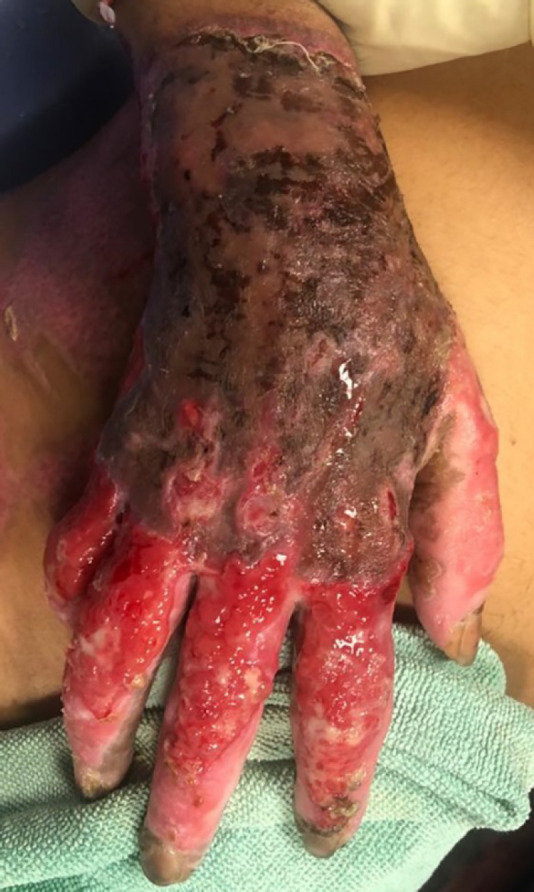
Case 2: Blisters and vesicles surrounded by erythema were observed in one finger (5^th^ April).

Morever, the topical potassium permanganate and vaseline gauze was applied once daily for dressing. In the physical examination during changing dress, blisters and vesicles surrounded by erythema were observed in one finger 14 days after hospitalization (April 5, 2020), so acyclovir was started (QID) (Figure 3). Following the treatment, targocid injection was begun daily from April 10, 2020. Evaluation of vital signs showed normal range and age (T: 37.2℃, RR: 20/mim, BP: 120/90 mmHg and PR: 120/mim) and the wound culture did not show any aerobic bacterial infection on the day of vesicular appearance (April 5, 2020). 

Due to the nature of burn injuries, these victims were susceptible to pathogenic microorganisms, so a set of diagnostic tests were implemented for the mentioned case to identify the vesicular cause as follows: Tzanck test for initial evaluation and pathological examination for HSV infection. Complementary tests such as qRT-PCR were performed for HSV, chicken pox, and COVID-19, and KOH for fungal infections. The results were negative for all of the above tests. It is worth to note that IgM and IgG values were 0.1 and 5.5, respectively for HSV denoting to her immunity against HSV. 

Based on hospitalization time during pandemic COVID-19 and dispel of any misidentification, NP/OP samples were taken for COVID-19 similar to the case 1 condition, 3 days after the vesicular appearance (April 11, 2020). The molecular test results for NP/OP samples were negative. Based on the radiology findings (chest CT scan), no evidence of COVID-19 was seen on images (April 13, 2020). The chest wall and diaphragm were intact and osseous structures were unremarkable. Six days later, she experienced fever of 38.4℃ (from April 19, 2020). Along with routine serial laboratory tests and standard treatment, debridement of the wounds with skin graft for face, thigh and hands were performed (April 20, 2020). 

For further evaluation and appropriate treatment, a few specimens were taken from the vesicular lesions with a sterile needle (one needle-one sample) for COVID-19 molecular assay (April 23, 2020). The results for these samples were all positive and confirmed SARS-COV-2. After 3 days (April 26, 2020), vesicular blisters appeared in areas other than burn wounds. From the first day of skin vesicular manifestation until now, topical tetracycline was used for her (Figure 3). The laboratory tests were summarized before and after appearance of vesicles in Table 2. Altogether the patient was hospitalized for 35 days (April 26, 2020). 

From bullae and surrounding areas, multiple punch biopsies were prepared. Hematoxylin and eosin slides were examined under light microscopy that showed intact epidermis without any viral inclusions or multinucleated cells (like cells in *Herpes simplex *virus or *Varicella zoster *virus infections), and there was subepidermal bullae containing RBCs and a few lymphocytes and rarely PMN leukocytes. Dermis revealed infiltration of lymphocytes and a few PMN leukocytes, as well as increase in fibroblasts and small vessels that were congested (like granulation tissue). Some tissue fragments illustrated ulceration and lymphocyte rich granulation tissue in the underlying dermis (Figure 2). 

## DISCUSSION

COVID-19 is a novel Corona virus pneumonia which was first reported in Wuhan and has drawn intense attention around the world. Over time, this disease is more spread worldwide and infects different groups of healthy and sick individuals with different underlying diseases, which causes different aspects of this disease to be identified. Although there are some reports on skin manifestations of COVID-19 around the world, this is the first report of skin vesicular manifestation in the burn victims, related to this pandemic infection.^7^^,^^8^

Due to the nature of the CoVID-19, which is known as a respiratory infection, in most medical centers, individuals are screened for shortness of breath, fever, cough, muscle pain, headache, and new loss of taste or smell.^9^ Although these are the main manifestation of this pandemic infection, some other unusual symptoms are appearing too. One of these unusual symptoms that have been seen in our studied cases is vesicular manifestation in burn patients, who are immunocompromised due to the loss of their skin. Some other unusual symptoms which are present and reported rarely during the COVID-19 crisis are nausea, diarrhea, and delirium, which may also be increased in continue.^10^

Also, in mentioned articles, the authors suggested that papulovesicular eruptions may possibly be associated with COVID-19, but at the same time, they believe that their data do not prove that this rash is caused by or definitively associated with COVID-19. However, the data obtained in our case study indicated a high probability of developing COVID-19 in burn patients with the manifestation of papulovesicular eruptions.^7^^,^^8^ Also, in some studies, cutaneous manifestations were reported to be associated with COVID-19, but according to the lack of iconographic and histological documentation, they were not confirmed, while the vesicles in our study were checked by histopathology and molecular tests. The important point in our study was that our cases had no fever for a few consecutive days, which was febrile rash in SARS-CoV-2 related infection, although having higher temperature is a usual matter for burn patients due to nature of disease.^11^

The remarkable topics in these two cases were controversy results for liver test in comparison with COVID-19 patients. In was revealed that in recent pandemic of our burn cases, ALT and AST had increasing rate, while the mentioned biomarkers decreased over hospitalization. One reason which may justify this occurrence is losing a huge volume of liquid in burn injury including liver injury as a common complication in these victims.^12^ In terms of laboratory inspection of cases, it was found that some biomarkers had similar patterns with other COVID-19 patients. Decreasing WBCs, hemoglobin, serum creatinine, and positive results of CRP revealed that appearance of vesicular lesions may accompany bacterial infection and immune deficiency is unfavorable prognosis in these two cases.^13^^,^^14^

Although much of the recent study’s evidence suggests that the COVID-19 may play an important role in the eruption of skin vesicles in burn patients, it is important to note that the potential side effects of the concomitant use of antibiotics may also play a role in vesicle formation suggesting further studies. Despite managing the patients and wards carefully and correctly to prevent epidemic of the virus in our burn center and cancelling the elective surgeries, unfortunately an overwhelming of disease was seen in our center. In general, the uncommon symptoms of cases gave early warring for other burn victims too. Any hospitalized patient or outpatient in burn departments should be considered as a potential infectious source of COVID-19 which may cause epidemic in burn wards and transmission by patients, nurses, physicians and other health care staffs. In initial evaluation of new patients, further precautions are an urgent need. Personal protective equipment and careful screening would be helpful in reducing the transition rate. In view of the fact that there are many and complicated staffs in the department, and some staffs need to take public transportation to get to work, it is suggested to establish an active health monitoring and reporting mechanism for the staffs.^15^^,^^16^

## CONCLUSION

In summary, the strict management of the burn ward is an important measure for epidemic prevention and control, especially in the case of a large number of emergency patients with burns. It is necessary to strengthen the protection and isolation of medical staffs, patients, and family members, so as to prevent the spread of the epidemic in the burn wards.
